# A Histological Study of Ovarian Development in the Giant Red Shrimp *Aristaeomorpha foliacea* (Crustacea: Decapoda: Aristeidae) from the Southern Tyrrhenian Sea (Western Mediterranean)

**DOI:** 10.1100/2012/289608

**Published:** 2012-04-26

**Authors:** Anna Perdichizzi, Laura Pirrera, Valeria Micale, Ugo Muglia, Paola Rinelli

**Affiliations:** ^1^National Research Council, Institute for Coastal Marine Environment (IAMC-CNR), Spianata S. Raineri 86, 98122 Messina, Italy; ^2^Department of Morphology, Biochemistry, Physiology and Animal Production, Faculty of Veterinary Medicine, University of Messina, Polo Universitario Annunziata, 98168 Messina, Italy

## Abstract

The reproductive features of the giant red shrimp, *Aristaeomorpha foliacea*, were investigated in the southern Tyrrhenian sea by experimental trawl sampling. The annual length-frequency distribution showed a multimodal trend in females, ranging between 16 and 67 mm carapace length (CL), and a unimodal trend in males (18–45 mm CL). Mature males occurred in different proportions all year round, while females displayed seasonal maturity (June—September), with a peak in July. Six oocyte developmental stages were identified, the most advanced of which (Pv, postvitellogenic) had never been described before in this species. Ovary development followed a group-synchronous pattern, with the yolked oocyte stock clearly separated from the reservoir of unyolked oocytes, suggesting that *A. foliacea* is a total spawner, with determinate fecundity. Based upon histological findings, a revision of macroscopic maturity staging employed in Mediterranean bottom trawl surveys (MEDITS) is proposed.

## 1. Introduction

The giant red shrimp *Aristaeomorpha foliacea* (Risso, 1827) (Decapoda, Aristeidae) is a widespread decapod crustacean in the eastern and western Atlantic, western Pacific, the Indian Ocean, as well as the Mediterranean Sea, showing a preference for muddy bottoms, with aggregation mainly in submarine trenches and canyons along the continental slope from 150 m to 1850 m with a peak in abundance between 300 m and 700 m [1, 2].

Together with the other red shrimp *Aristeus antennatus* (Risso, 1816), *A. foliacea* represents an important target species of commercial fleet fishing off the southern Tyrrhenian Sea operating over the continental slope, with total landings of the order of one hundred tons [3]. Because of its economic value, many studies have been carried out on the biology, population ecology, and fisheries of this shrimp [4–13].

Reproductive biology is one of the main concerns in formulating proper management practices for fishery science. In fact, the relationship between size and reproduction events, such as mating and sexual maturity, is crucial to understand the dynamics of a stock. Knowledge of reproductive biology of *A. foliacea* has mainly come from studies on ovary maturation of fresh specimens by means of macroscopical investigations [10, 13–19].

On the other hand, few studies examined histology of ovarian development [20–23], and many aspects, such as the spawning pattern and type of fecundity, remain to be solved. International commissions for fisheries study (i.e., ICES, International Council for the Exploration of the Sea, GFCM, General Fisheries Council for the Mediterranean) strongly recommend standardizing criteria for evaluating sexual maturity in target species. Recently, the need for a common system for classification of maturity stages in Crustaceans has been highlighted by an Expert Group of ICES (2010), which also pointed out the importance of histology as a tool for obtaining the highest accuracy in these studies.

 This paper describes for the first time some reproductive features of *A. foliacea* caught in the southern Tyrrhenian Sea, such as monthly maturity stages by size and length structures by maturity stage. Moreover histological analyses of ovaries were performed in order to validate the empirical, macroscopic scale used in experimental trawl surveys through a detailed description of maturity stages based upon oocyte development. The information provided by this paper is intended to contribute to establishing production and population dynamics models, for a rational exploitation of this important fishery resource.

## 2. Material and Methods

### 2.1. Biological Data and Reproductive Parameters

**Figure 1:**
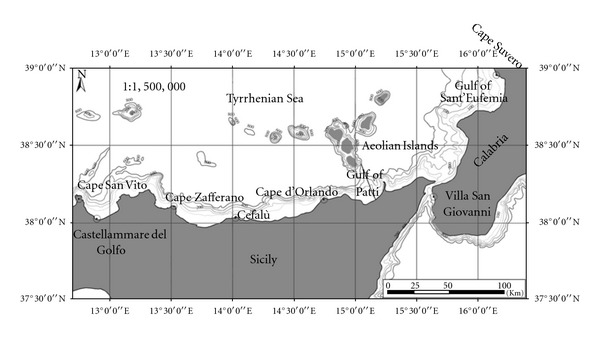
Sampling area.

Red shrimps were caught on a monthly basis (September 2008 through August 2009) by commercial trawlers operating in the southern Tyrrhenian Sea, between the Gulf of Patti and Aeolian Islands, in a depth range from 500 to 700 metres (Figure 1). The sampling was conducted in order to sample all stages of maturity. Specimens (*n* = 21 163) were sexed and measured for carapace length (CL) to the nearest of 0.01 mm. The overall length frequency distributions (LFDs) of males and females for all year were estimated. The differences between the two sexes were tested by Kolmogorov-Smirnov test (*P* ≤ 0.001). Body weight (BW) was recorded to the nearest 0.01 g. Gonad weight (GW) of females was recorded to the nearest 0.001 g, and gonadosomatic index (GSI) was estimated as (GW/BW) × 100. 

On the basis of the colour and shape of the ovary and testis, each specimen was classified according to the macroscopic maturity scale adopted in MEDITS programme (Table 1). Males maturity was checked by the presence of the spermatophores in the terminal ampoullae of vasa deferentia. Presence or absence of spermatophores in the thelycum of females (inseminated specimens) was also recorded. 

The length structures by maturity stage were estimated by analyzing the box-plot representation. Statistical differences between maturity stages and carapace length were tested by Kruskal-Wallis test (*P* ≤ 0.001). 

### 2.2. Ovarian Histology

At least 10 females of each maturity stage were fixed on board in 10% neutral formalin, after cutting the integument. Once in the laboratory, after washing in running tap water for 4 hours, ovaries were dissected, dehydrated in a graded series of ethanol, embedded in resin, and cut in 5 *μ*m thick sections. Sections were stained with haematoxylin-eosin and observed under a light microscope for histological description of oocyte development and ovarian maturation. Oocyte size was estimated by the imaging software Leica IM1000 v 1.20, by taking the average between the major and minor axis in rounded oocytes, and by measuring the major axis (length) of rectangular oocytes, for the sake of comparison with previous studies [23].

## 3. Results

### 3.1. Population Structure

**Figure 2:**
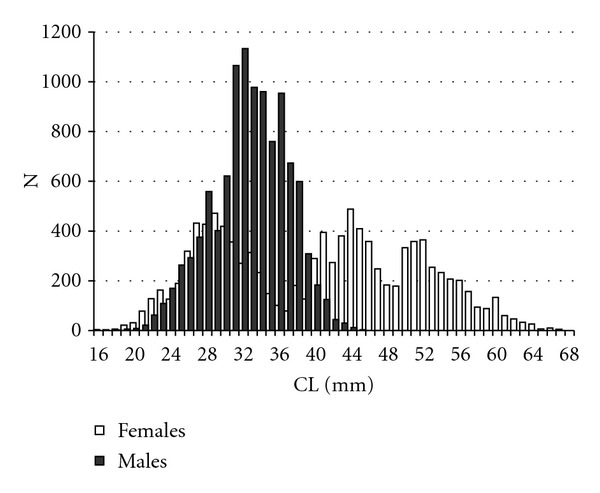
Length frequency distributions (LFDs) of females and males of* Aristaeomorpha foliacea* caught in the southern Tyrrhenian Sea.

The population structure of females and males is presented in Figure 2. The size of females ranged from 16 to 67 mm CL, showing a multimodal trend during the year. The most abundant individuals had carapace length generally between 27 and 30 mm. 

 The males measured between 18 and 45 mm CL and exhibited a unimodal distribution. A significant difference in LFD between males and females was found (*P* < 0.001).

### 3.2. Gonadal Maturation

**Figure 3:**
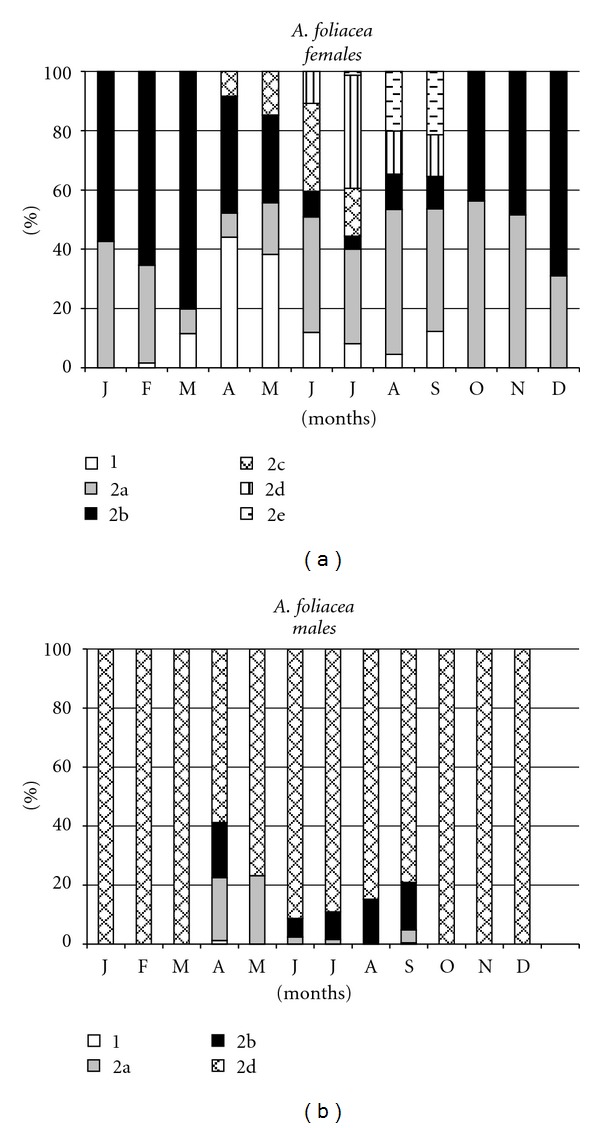
Monthly occurrence of ovarian maturity stages of females (a) and males (b) of *Aristaeomorpha foliacea* from the southern Tyrrhenian Sea.

Females of *Aristaeomorpha foliacea* displayed seasonal maturity. Ovarian maturation started in April (stage 2c = 8%). Mature females (stage 2d) were found from June through September, with a peak value in July (2d = 38%) (Figure 3(a)). Spent individuals appeared in August (20.1%) and September (21.3%). Females with stages 2a (developing virgin) and 2b (recovering) ovaries were present throughout the year, while specimens with immature ovaries (stage 1) were caught from February to September. 

The smallest female with mature ovaries (stage 2d) was observed in July and measured 41 mm CL. Only larger females (CL range: 41–66 mm) contributed to the reproductive pool, which, taking into consideration both maturing (2c) and mature (2d) specimens, represented about 50% of total during the spawning peak. 

 The occurrence of reproductive males of *A. foliacea*, characterized by the presence of spermatophores in the terminal ampullae, was constant and very high during the whole sampling period, with values ranging from 59% to 100% (Figure 3(b)). The size range of mature males ranged from 25 to 45 mm CL. 

The size at maturity stage analysis, made on all combined months, indicated, for both sexes, a satisfactory resolution capability of the adopted scale. For females the 2a stage prevailed (36.7%) while for males the 2d stage prevailed (90.4%) (Table 2). 

**Figure 4:**
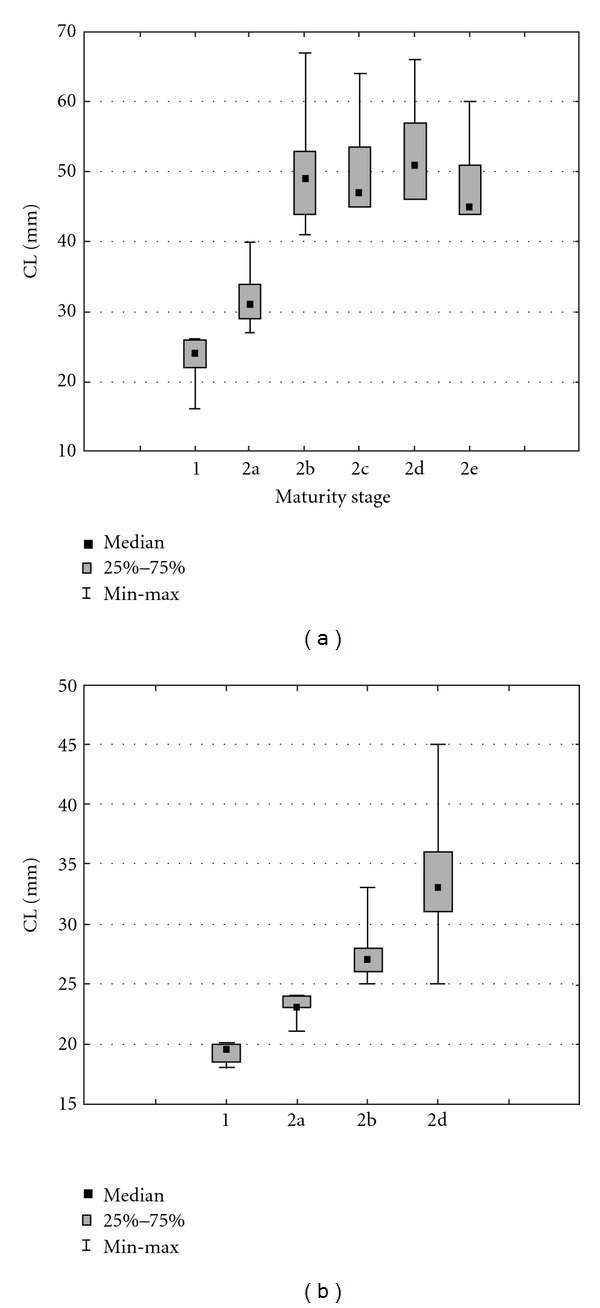
Overall (all months combined) box-plot representation of length structure by sex (females, (a) and males, (b)) and maturity stage in *Aristaeomorpha foliacea. *

 Relation between median and maturity stages showed significant differences for both sexes (*P* < 0.001), except between stages 2b and 2c (*P* = 0.22) and stages 2b and 2e (*P* = 0.30) for females (Figure 4).

### 3.3. Histology of Ovary Development

**Figure 5:**
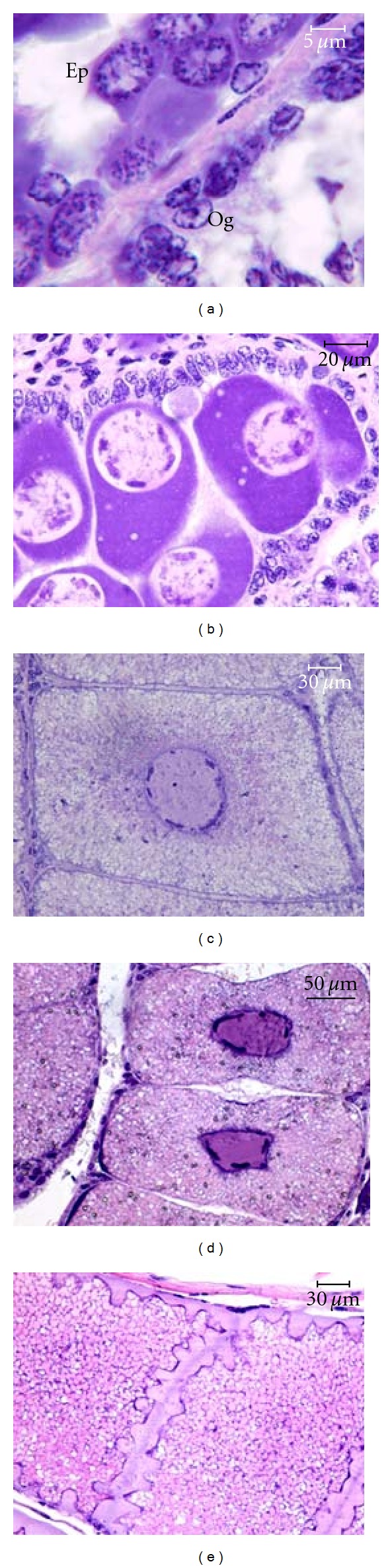
Oocyte developmental stages of *Aristaeomorpha foliacea*. (a) Oogonia (Og) and early primary stage (Ep); (b) late primary stage; (c) early vitellogenic stage; (d) late vitellogenic stage; (e) post-vitellogenic stage. (Haematoxylin-eosin.)

**Figure 6:**
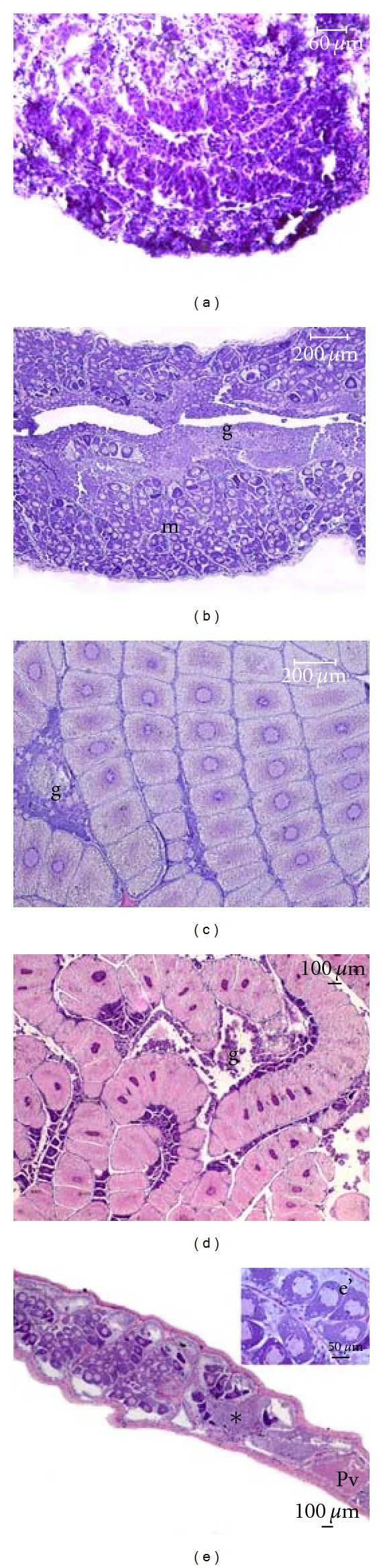
Ovarian maturity stages of *Aristaeomorpha foliacea* (haematoxylin-eosin). (a) Immature ovary (stage 1); (b) recovering ovary (stage 2b), showing distinct germinative (g) and maturative (m) zones, the latter populated by late primary oocytes organized into tubule-like structural units. (c) Maturing ovary (stage 2c) populated by early vitellogenic oocytes, among which small germinative islets (g) can be seen. (d) Mature ovary (stage 2d) populated by late vitellogenic oocytes creating a mosaic structure. g: germinative islets. (e) Spent ovary (stage 2e) containing a residual post-vitellogenic oocyte (Pv), besides degenerating late primary oocytes displaying severe vacuolization of the cytoplasm (e'). Proliferation and hypertrophy of mesodermal cells (asterisk) are also evident.

Six oocyte developmental stages could be distinguished from the histological analysis of ovaries (Figure 5), namely, oogonia (Oo), early primary oocytes (Ep), late primary oocytes (Lp), early vitellogenic oocytes (Ev), late vitellogenic oocytes (Lv), and postvitellogenic oocytes (Pv), whose microscopic features are synthetically reported in Table 3. Post-vitellogenic oocytes, which have never been reported so far in *A. foliacea*, were found in some specimens macroscopically staged 2d (mature) (Table 1). These oocytes had the same size and yolk inclusions as the late vitellogenic ones (350–470 *μ*m), but their nucleus was not visible, and they displayed slightly eosinophilic, columnar *cristae* protruding into the outer cytoplasmic cortex (Figure 5(e)), strongly resembling penaeid cortical rods [24, 25]. For these reasons, such oocytes were considered to have achieved vitellogenesis and started final maturation prior to ovulation. Another type of oocyte was found, that is, Lp oocytes, showing massive vacuolization of cytoplasm (Figure 6(e)). As these oocytes occurred only in spent specimens, they are likely to be abortive oocytes at an initial stage of atresia (Ao), destined to be resorbed. 

The histological description of macroscopic maturity stages employed in MEDITS surveys is reported in Table 4 and Figure 6. Immature ovaries (stage 1) showed an even connective stroma populated by oogonia, early primary oocytes, and occasional late primary oocytes. In all other specimens, the ovarian parenchyma consisted of germinal and maturative zones, the former containing oogonia and early primary oocytes and the latter containing later oocyte stages. Stage 2a (virgin developing) and 2b (recovering) ovaries were histologically identical, with Lp oocytes as the most advanced developmental stage. As ovary maturation proceeded, developing oocytes occupied most of the ovarian parenchyma, leaving only small germinative islets of resting oocytes interspersed between them. Oocyte development in the maturative parenchyma occurred in a synchronous way, as indicated by a single batch of oocytes at the early vitellogenic (Ev) stage in maturing (2c) ovaries and a single batch of oocytes at the late vitellogenic (Lv) or post-vitellogenic (Pv) stage in mature (2d) ovaries. Resting (2e) ovaries were characterized by hyperaemia of ovarian stroma and marked proliferation and hypertrophy of mesodermal (somatic) cells lining tubular units. The latter were populated by atresic oocytes (Ao), among which some residual Pv oocytes could be detected. 

The lowest and highest GSI values of females examined in the present study were 0.006 (stage 1 specimen with CL = 25 mm) and 13.87 (stage 2d specimen with CL = 46 mm), respectively. The mean GSI values for each maturity stage are reported in Table 4.

## 4. Discussion

The giant red shrimp, *Aristaeomorpha foliacea*, is among the most prized demersal resources exploited by deep-sea fishery in the southern Tyrrhenian Sea. The importance of this resource to Mediterranean fisheries is supported by the results on the biology and ecology of this species, obtained from more than twenty years of trawl survey programmes (i.e., GRUND, Valutazione delle Risorse Demersali, and MEDITS, *International Bottom* Trawl Survey in the *Mediterranean*). 

 The reproductive season of *A. foliacea* in the studied area, as indicated by presence of mature females (June–September), is comparable with that one reported along the Sardinian coasts [26], but it is shorter than in northern areas of western Mediterranean [16, 20], as well as than in eastern Mediterranean [18, 19]. On the other hand, the occurrence of mature males (≥50%) in all months, suggesting no evidence of a seasonal maturity cycle, seems to be a constant reproductive feature of *A. foliacea* all over the Mediterranean. Such a protracted reproductive capacity of males could be related to mating activity. In fact, inseminated females occurred all year round, as in all Mediterranean populations studied [16–19]. Inseminated females were staged 2b through 2e, suggesting that mating may take place also after oviposition. On the other hand, immature (stage 1) and developing virgins (stage 2a) were never inseminated, suggesting that ovary maturation is triggered by mating. In other areas, resting females bearing spermatophores were reported in proportions ranging from 6% (west Pacific) [22] to 60% (eastern Mediterranean) [19]. However, while ovary maturation has been reported to follow mating by four months in eastern Mediterranean [19], it would seem that it occurs within one month from mating during the first reproductive cycle in our western Mediterranean population. The lower proportion of mature males from April to September was not paralleled by a decreased number of inseminated females in the same months, suggesting the possibility that males are able to mate with multiple females, as hypothesized in *Aristeus antennatus* [19]. 

 The fully mature female minimum size of *A. foliacea* in the present study (41 mm CL) is by far the largest ever recorded in the Mediterranean (27–40 mm CL), confirming a westward gradient in minimum size at maturity [18]. The size at maturity stage analysis, made on all combined months, indicated, for both sexes, a satisfactory resolution capability of the adopted scale. 

 A reliable maturity staging is needed for a correct evaluation of spawning-stock biomass (SSB) (i.e., the biomass of reproducing individuals in a population), which is crucial in stock assessment studies. In fact, it has been demonstrated that macroscopic staging of the gonads may lead to overestimating SSB by up to 35% in fish [27]. For this reason, histological analysis of gonads is recommended to validate macroscopic observations, as well as to standardize criteria for comparing maturity data from different populations [28]. This is particularly true for crustacean species, whose reproductive features are less documented than those of fish. 

 The GSI values of the different maturity stages were similar to those from other studies, except for the immature stage, which was considerably lower than those reported not only in Taiwan and eastern Mediterranean [19, 22], but also in a population of western Mediterranean [21]. 

 The histological observation of ovaries in *A. foliacea* allowed us to detect a novel oogenic stage, which had not been described in previous studies [21–23], namely, the post-vitellogenic stage. Such stage was characterized by the presence of cortical cristae, resembling the “cortical rods” of penaeid shrimps [29, 30]. Cortical rods are formed after completion of yolk accumulation and are involved in the cortical reaction initiated by contact with sea water [29], by forming a jelly layer around the egg, preventing polyspermy. In penaeid oocytes, which possess a vitelline envelope, cortical rods develop outside the oocyte plasma membrane but under the vitelline membrane [30]. In our study, light microscopy showed that cortical *cristae* are housed in crypts formed by invaginations of the oolemma, and therefore they are extracellular, as cortical rods. 

 Ovary maturation in *A. foliacea* involved a large batch of oocytes developing synchronously inside tubular units, with resting oocytes remaining in the germinative islets between them. Prespawning ovaries were almost completely filled with post-vitellogenic oocytes, to be released at the next spawning, while oogonia and oocytes at the previtellogenic stages remained in the ovary, with the most advanced of them (Lp oocytes) undergoing degeneration after spawning. Such features are indicative of a group-synchronous ovarian developmental pattern [31] and strongly suggest that *A. foliacea* has a determinate fecundity, that is, fecundity is fixed before the onset of spawning [32]. It is likely that *A. foliacea* is a total spawner, that is, all yolked oocytes are released in a unique spawning event during each reproductive season, as *A. antennatus* [33]. 

 For the first time, the histological features of spent ovaries were described. In these ovaries, which was found in August and September, the architecture of ovarian stroma appeared modified, by proliferation and hypertrophy of mesodermal cells, accompanied by hyperaemia, while pre-vitellogenic oocytes (Lp stage) left in the ovary after spawning of mature eggs displayed severe vacuolization of the cytoplasm. This is likely to be an initial phase of atresia, suggesting future resorption of these oocytes, while the oocytes destined to develop in the next reproductive season may be recruited from the batch of early primary oocytes left in the ovary. The occurrence of the “spent” stage in *A. foliacea* has been debated, since the first observations [21], which lead [22] to hypothesize that females may die after spawning, or move to waters deeper than 600 m, where they may be missed by trawlers. On the other hand, [19] reported 16% spent specimens from July, consistently with the 1-month-advanced spawning in that area. Our results suggest that *A. foliacea* starts reorganizing the ovary soon after spawning, and this process lasts about 2 months, after which a recovering ovary with no signs of previous spawning or oocyte resorption is found. 

 On the basis of histological observations, a reduction of the macroscopic maturity stages employed in MEDITS survey to five (namely, (1) immature, (2) developing/recovering, (3) maturing, (4) mature, and (5) spent) could be advisable, as proposed in the framework of the Workshop on crustacean (*Aristeus antennatus*, *Aristaeomorpha foliacea*, *Parapenaeus longirostris*, *Nephrops norvegicus*) maturity stages (WKMSC) held in Messina (Italy) in 2009 [28].

## Figures and Tables

**Table 1:** Macroscopic maturity scale adopted in MEDITS trawl surveys (MEDITS Instruction Manual v.5.0, 2008).

*Sex *	*Reproductive apparatus aspect *	*Colouring of fresh ovary *	*Maturation state *	Stage
U	Sex not distinguished by naked eye. Sex undetermined	translucid	**Undetermined **	0

F	Ovary hardy visible in transparence. After dissection of the tegument ovary is small and lobes are flaccid, stringy and poorly developed. No sphermatophores on thelycum.	Whitish or translucid	Immature = virgin*	1
M	Petasma is not much visible, and there is not spermatic masses (emi-spermatophores) on the seminal ampullae, located on side of the V pair of pereiopods. Long rostrum.			

F	Ovary status to develop. Cephalic and lateral lobes are small but distinguishable with the naked eye. Abdominal extension are thin and just visible.	Flesh coloured	Virgin developing**	2a
M	Petasma appears visible and nearly or completely joined, but there are no spermatic masses in the seminar ampullae. Long or intermediate rostrum.			

F	Ovary status to redevelop. Cephalic and lateral lobes are small but distinguishable by naked eye. Abdominal extension is thin and just visible. Occasionally presence of spermatophores.	Flesh coloured	Recovering**	2b
M	Petasma appears completely joined, but there are no spermatic masses in the seminar ampullae. Short rostrum.			

F	Ovary developed and occupies almost entirely the dorsal portion. The cephalic and lateral lobes are much developed and have a turgid consistence.	Light and dark grey	** Maturing or almost mature**	2c
M				

F	Turgid ovary extends to the whole dorsal portion, covering the organs below. Lobes and extensions well developed, in particular the abdominal extension is much evident. Oocytes well visible.	Black	** Mature **	2d
M	Petasma is perfectly visible and completely joined. Spermatic masses in seminar ampullae. Small rostrum.			

F	Resting ovary. Presence of spermatophores.	Uncoloured	Resting adult*	2e

**Adult specimens.**

*, **: *Warning*! Be careful. These stages could be confused with each other.

**Table 2:** Length composition characterization of *Aristaeomorpha foliacea *according to the box plot approach, by stage, sex, and years combined.

Sex	Stage	*n*	Mean	Median	Minimum	Maximum	Lower quartile	Higher quartile	sd
Females	1	1 069	23.83	24	16	26	22	26	2.08
	2a	3 835	31.93	31	27	40	29	34	3.97
	2b	3 507	48.78	49	41	67	44	53	5.61
	2c	558	49.38	47	41	64	45	53.5	5.73
	2d	849	51.80	51	41	66	46	57	6.35
	2e	620	47.56	45	41	60	44	51	5.34

Males	1	16	19.25	19.5	18	20	19	20	0.86
	2a	363	23.18	23	21	24	23	24	0.92
	2b	647	27.17	27	25	33	26	28	1.71
	2d	9 699	33.40	33	25	45	31	36	3.60

**Table 3:** Histological description of oocyte developmental stages in *Aristaeomorpha foliacea. *

Oogenic stage		Size (*μ*m)	Histological description
	Oogonia (Og)	<10	Large, clear nucleus containing many strongly basophilic nucleoli, one of which is more evident. Cytoplasm barely visible.
	Early primary (Ep)	15–25	Large, clear nucleus containing chromatin clusters and filaments. Cytoplasm barely visible.
	Late primary (Lp)	25–85	Large, clear nucleus containing many peripheral nucleoli. Strongly basophilic cytoplasm.
Oocytes	Early vitellogenic (Ev)	90–300	Rectangular in section. Round, central nucleus, with flattened nucleoli leaned against the nuclear envelope. Cytoplasm filled with lipid vesicles. Eosinophilic, yolk granules form a ring around the nucleus.
	Late vitellogenic (Lv)	200–350	Rectangular in section. Small, central nucleus. Cytoplasm filled with lipid vesicles and yolk granules.
	Post-vitellogenic (Pv)	350–470	Nucleus no longer visible. Cytoplasm filled with yolk granules and lipid vesicles. Slightly eosinophilic, columnar *cristae* protruding into the outer cytoplasmic cortex.
	Atresic (Ao)	—	As Lp oocytes, showing massive vacuolization of cytoplasm.

**Table 4:** Histological description of ovarian maturity stages in *Aristaeomorpha foliacea*, with their relative GSI (mean ± s.d.).

Maturity stage		Histological description	GSI
(MEDITS Instruction Manual v.5.0, 2008)			
1	Immature	Oogonia (Og) and early primary oocytes (Ep) scattered in the connective stroma. Late primary oocytes (Lp) occasionally present.	0.11 ± 0.08 (*n* = 11)
2a	Virgin developing	Germinative zone containing Oo and Ep. Maturative zone containing Lp, organized into tubule-like structural units.	0.53 ± 0.13 (*n* = 10)
2b	Recovering	The same as 2a.	1.17 ± 0.69 (*n* = 59)
2c	Maturing	Maturative parenchyma containing a single batch of early vitellogenic (Ev) oocytes, organized into tubule-like structural units. Germinative islets populated by Oo and Ep oocytes.	5.07 ± 1.64 (*n* = 14)
2d	Mature	Maturative parenchyma containing late vitellogenic oocytes (Lv), piled up in tubular units. The most advanced specimens contain post-vitellogenic oocytes (Pv). Germinative islets populated by Oo and Ep oocytes.	9.29 ± 2.51 (*n* = 11)
2e	Resting	Proliferation and hypertrophy of mesodermal cells lining tubular units. The latter are populated by Lp oocytes at an initial stage of atresia (Ao), among which some residual Pv oocyte could be detected.	2.17 ± 0.47 (*n* = 9)

## References

[1] Bianchini ML, Ragonese S Life cycles and fisheries of the deep-water red shrimps *Aristaeomorpha foliacea* and *Aristeus antennatus*.

[2] Pérez Farfante I, Kensley B (1997). *Penaeoid and Sergestoid Shrimps and Prawns of the World: Keys and Diagnoses for the Families and Genera*.

[3] IREPA Istituto Ricerche Economiche per la Pesca (2008). *Osservatorio Economico Sulle Strutture Produttive Della Pesca Marittima in Italia 2008*.

[4] Brian A (1931). La biologia del fondo a ‘Scampi’ nel Mar ligure. *5.Aristaeomorpha, Aristeus ed altri Macruri reptanti Bollettino dei Musei di Zoologia e Anatomia Comparata della R*.

[5] Holthuis LB (1980). *Shrimps and Lobsters of the World: An Annotated Catalogue of Species of Interest to Fisheries Volume 1 of FAO Species Catalogue*.

[6] Cau A, Deiana AM, Mura M (1987). Ecological observations on *Aristaeomorpha foliacea* (Risso, 1827) (Decapoda, Penaeidae) in the mid-Western Mediterranean Sea. *Investigacion Pesquera*.

[7] Ragonese S, Bianchini ML, Gallucci VF (1994). Growth and mortality of the red shrimp *Aristaeomorpha foliacea* in the Sicilian Channel (Mediterranean Sea). *Crustaceana*.

[8] Spedicato MT, Lembo G, Carbonara P, Silecchia T, Bianchini ML, Ragonese S (1994). Biological parameters and dynamics of *Aristaeomorpha foliacea* in Southern Tyrrhenian Sea. *Life Cycles and Fisheries of the Deep-Water Red Shrimps Aristaeomorpha Foliacea and Aristeus Antennatus*.

[9] Ragonese S Geographical distribution of *Aristaeomorpha foliacea* (Crustacea, Aristeidae) in the Sicilian Channel (Mediterranean Sea).

[10] Ragonese S, Bianchini ML (1995). Size at sexual maturity in red shrimp females, *Aristaeomorpha foliacea*, from the Sicilian Channel (Mediterranean Sea). *Crustaceana*.

[11] Matarrese A, D’onghia G, DE Florio M, Panza M, Costantino G (1995). Recenti acquisizioni sulla distribuzione batimetrica di *Aristaeomorpha foliacea* ed *Aristeus antennatus* (Crustacea, Decapoda) nel Mar Ionio. *Biologia Marina Mediterranea*.

[12] Matarrese A, D'onghia G, Tursi A, Maiorano P Vulnerabilità e resilienza di *Aristaeomorpha foliacea* (Risso, 1827) e *Aristeus antennatus* (Risso, 1816) (Crostacei, Decapodi) nel Mar Ionio.

[13] D’onghia G, Maiorano P, Matarrese A, Tursi A (1998). Distribution, biology, and population dynamics of *Aristaeomorpha foliacea* (Risso, 1827) (Decapoda, Natantia, Aristeidae) in the north-western Ionian Sea (Mediterranean Sea). *Crustaceana*.

[14] Mura M, Campisi S, Cau A (1992). Osservazioni sulla biologia riproduttiva negli aristeidi demersali del Mediterraneo Centro-Occidentale. *Oebalia*.

[15] Mori M, Biagi F, DE Ranieri S, Bianchini ML, Ragonese S (1994). Reproductive biology of *Aristaeomorpha foliacea* in the Southern Tuscany archipelago (Central Tyrrhenian Sea). *Life Cycles and Fisheries of the Deep-water Red Shrimps Aristaeomorpha foliacea and Aristeus antennatus*.

[16] Belcari P, Viva C, Mori M, De Ranieri S (2003). Fishery and biology of *Aristaeomorpha foliacea* (Risso, 1827) (Crustacea: Decapoda) in the northern Tyrrhenian Sea (Western Mediterranean). *Journal of Northwest Atlantic Fishery Science*.

[17] Papaconstantinou C, Kapiris K (2003). The biology of the giant red shrimp (*Aristaeomorpha foliacea*) at an unexploited fishing ground in the Greek Ionian Sea. *Fisheries Research*.

[18] Politou CY, Kapiris K, Maiorano P, Capezzuto F, Dokos J (2004). Deep-sea Mediterranean biology: the case of *Aristaeomorpha foliacea* (Risso, 1827) (Crustacea: Decapoda: Aristeidae). *Scientia Marina*.

[19] Kapiris K, Thessalou-legaki M (2009). Comparative reproduction aspects of the deep-water shrimps *Aristaeomorpha foliacea* and *Aristeus antennatus* (Decapoda, Aristeidae) in the Greek Ionian Sea (Eastern Mediterranean). *Journal of Zoology Article*.

[20] Orsi Relini L, Semeria M (1983). Oogenesis and fecundity in bathyal penaeid prawns, *Aristeus antennatus* and *Aristaeomorpha foliacea*. *Rapport de la Commission Internationale pour la Exploration Scientifique de la Mer Mediterranée*.

[21] Levi D, Vacchi M (1988). Macroscopic scale for simple and rapid determination of sexual maturity in *Aristaeomorpha foliacea* (Risso, 1826) (Decapoda: Penaeidae). *Journal of Crustacean*.

[22] Kao HC, Chan TY, Yu HP (1999). Ovary development of the deep-water shrimp *Aristaeomorpha foliacea* (Risso, 1826) (Crustacea: Decapoda: Aristeidae) from Taiwan. *Zoological Studies*.

[23] Desantis S, Labate M, Maiorano P, Tursi A, Labate GM, Ciccarelli M (2001). A histochemical and ultrastructural study of oogenesis in *Aristaeomorpha foliacea* (Risso, 1827). *Hydrobiologia*.

[24] Yamano K, Qiu GF, Unuma T (2004). Molecular cloning and ovarian expression profiles of thrombospondin, a major component of cortical rods in mature oocytes of penaeid shrimp, *Marsupenaeus japonicus*. *Biology of Reproduction*.

[25] Peixoto S, Coman G, Arnold S, Crocos P, Preston N (2005). Histological examination of final oocyte maturation and atresia in wild and domesticated *Penaeus monodon* (Fabricius) broodstock. *Aquaculture Research*.

[26] Follesa MC, Cuccu D, Murenu M, Sabatini A, Cau A (1998). Aspetti riproduttivi negli Aristeidi, *Aristaemorpha foliacea* (Risso, 1827) e *Aristeus antennatus* (Risso, 1816), della classe di età 0^+^ e 1^+^. *Biologia Marina Mediterranea*.

[27] Vitale F, Svedäng H, Cardinale M (2006). Histological analysis invalidates macroscopically determined maturity ogives of the Kattegat cod (*Gadus morhua*) and suggests new proxies for estimating maturity status of individual fish. *ICES Journal of Marine Science*.

[28] ICES *Aristeus antennatus, Aristaeomorpha foliacea, Parapenaeus longirostris, Nephrops norvegicus* maturity stages.

[29] Clark WH, Lynn JW, Yudin AI, Persyn HO (1980). Morphology of the cortical region in the eggs of *Penaeus atzecus*. *Biological Bulletin*.

[30] Clark WH, Yudin AI, Lunn JW, Griffin FJ, Pillai MC (1990). Jellymlayer formation in penaeoidean shrimp eggs. *Biological Bulletin*.

[31] Wallace RA, Selman K (1981). Cellular and dynamic aspects of Oocyte growth in Teleosts. *Integrative and Comparative Biology*.

[32] Hunter JR, Macewicz BJ, Chyan-Huei Lo N, Kimbrell CA (1992). Fecundity, spawning, and maturity of female Dover sole *Microstomus pacificus*, with an evaluation of assumptions and precision. *Fishery Bulletin*.

[33] Carbonell A, Grau A, Lauronce V, Gómez C (2006). Ovary development of the red shrimp, *Aristeus antennatus* (Risso, 1816) from the northwestern Mediterranean Sea. *Crustaceana*.

